# Comparing hospital mortality – how to count does matter for patients hospitalized for acute myocardial infarction (AMI), stroke and hip fracture

**DOI:** 10.1186/1472-6963-12-364

**Published:** 2012-10-22

**Authors:** Doris T Kristoffersen, Jon Helgeland, Jocelyne Clench-Aas, Petter Laake, Marit B Veierød

**Affiliations:** 1 Norwegian Knowledge Centre for the Health Services, Quality Measurement Unit, PO Box 7004, St.Olavs plass, N-0130, Oslo, Norway; 2 Division of Mental Health, Norwegian Institute of Public Health, Oslo, Norway; 3 Department of Biostatistics, Institute of Basic Medical Sciences, University of Oslo, Oslo, Norway

**Keywords:** Mortality, Quality indicator, Transferred patients, AMI, Stroke, Hip fracture, Cause of death, Hospital comparison, Episode of care

## Abstract

**Background:**

Mortality is a widely used, but often criticised, quality indicator for hospitals. In many countries, mortality is calculated from in-hospital deaths, due to limited access to follow-up data on patients transferred between hospitals and on discharged patients. The objectives were to: i) summarize time, place and cause of death for first time acute myocardial infarction (AMI), stroke and hip fracture, ii) compare case-mix adjusted 30-day mortality measures based on in-hospital deaths and in-and-out-of hospital deaths, with and without patients transferred to other hospitals.

**Methods:**

Norwegian hospital data within a 5-year period were merged with information from official registers. Mortality based on in-and-out-of-hospital deaths, weighted according to length of stay at each hospital for transferred patients (W30D), was compared to a) mortality based on in-and-out-of-hospital deaths excluding patients treated at two or more hospitals (S30D), and b) mortality based on in-hospital deaths (IH30D). Adjusted mortalities were estimated by logistic regression which, in addition to hospital, included age, sex and stage of disease. The hospitals were assigned outlier status according to the Z-values for hospitals in the models; low mortality: Z-values below the 5-percentile, high mortality: Z-values above the 95-percentile, medium mortality: remaining hospitals.

**Results:**

The data included 48 048 AMI patients, 47 854 stroke patients and 40 142 hip fracture patients from 55, 59 and 58 hospitals, respectively. The overall relative frequencies of deaths within 30 days were 19.1% (AMI), 17.6% (stroke) and 7.8% (hip fracture). The cause of death diagnoses included the referral diagnosis for 73.8-89.6% of the deaths within 30 days. When comparing S30D versus W30D outlier status changed for 14.6% (AMI), 15.3% (stroke) and 36.2% (hip fracture) of the hospitals. For IH30D compared to W30D outlier status changed for 18.2% (AMI), 25.4% (stroke) and 27.6% (hip fracture) of the hospitals.

**Conclusions:**

Mortality measures based on in-hospital deaths alone, or measures excluding admissions for transferred patients, can be misleading as indicators of hospital performance. We propose to attribute the outcome to all hospitals by fraction of time spent in each hospital for patients transferred between hospitals to reduce bias due to double counting or exclusion of hospital stays.

## Background

Hospital quality indicators are utilized for the comparison of hospital performance and individual hospital monitoring as well as benchmarking health care services of provinces and countries [1-5]. A quality indicator based on patient outcomes has three essential elements: the medical diagnosis, the time to measured outcome (e.g. death, readmission, surgery), and the place of the outcome (e.g. hospital, home, institution). Mortality has been widely evaluated as a quality indicator [6-13].

Large variation in hospital ranking and outlier detection has been found when mortality measures were calculated by different methods [9,14-16]. An inherent problem with in-hospital mortality is that it reflects to a great degree hospital discharge practices [9,16]. Hospitals discharging patients early may seem to perform better than hospitals with longer patient stay. For patients treated at more than one hospital (transferred patients), the outcome should be attributed to all involved hospitals [13]. However, double-counting of patients may introduce bias [13,15].

A mortality-based indicator should include all-cause, in-and-out-of hospital deaths within a standardized follow-up period, e.g. 30 days. Data on in-hospital deaths is readily available, but obtaining data including out-of-hospital deaths and transfer information may be a challenge. Studies have found that for some medical conditions, the hospital profiles were similar when comparing mortality calculated from in-hospital deaths and in-and-out-of hospital deaths within 30 days (counting from start of admission, regardless of cause) [9,17]. Others report differences depending on time, place and cause of death included for the mortality measurement [10,15,16,18-20]. However, for transferred patients, previous studies have attributed the outcome to the first or the last hospital in the chain of admissions or used single-hospital stays only [16,18,19]. To our knowledge, no previous study has attributed the outcome to all involved hospitals without double counting.

First time acute myocardial infarction (AMI), stroke and hip fracture are three common, serious and resource-demanding medical conditions. They were selected by the Norwegian Directorate for Health and Social Affairs for developing mortality as a quality indicator for Norwegian hospitals [21]. All permanent residents in Norway have a personal identification number (PIN) which enables linking between hospital data and official registers*.* This offers a unique opportunity to compare mortality measures that differ with respect to time and place of death and to study the impact of transfers at the national level.

The objectives of the present work were to: i) summarize time, place and cause of death for patients hospitalized with AMI, stroke and hip fracture, ii) compare risk-adjusted mortality measures based on both in-hospital deaths and in-and-out-of- hospital deaths, with and without patients transferred to other hospitals.

## Methods

### Data sources

We collected data from all 66 Norwegian hospitals that had acute admissions of AMI, stroke and hip fracture during 1997–2001. The data sources were: the Patient Administrative System (PAS) of each hospital which provided type of admission (acute or elective), primary and secondary diagnoses, time and date of admission, and time and date of discharge; the National Population Register which provided age, gender, and date of death; the Norwegian Causes of Death Register which provided date and cause of death. An in-house developed data extraction system semi-automatically collected the PAS data in an encrypted format [21]. Statistics Norway prepared an encrypted PIN for linking the data sources.

The study protocol for the development and evaluation of 30D as a quality indicator for Norwegian hospitals was submitted to the Regional Ethical Committee. Because the project was a study of quality with the use of existing administrative data, ethical approval was not necessary and regarded by the Committee as outside their mandate. The use of data was approved by the Norwegian Data Inspectorate and the Ministry of Health.

### Inclusion and exclusion criteria

PAS records for AMI, stroke and hip fracture at each hospital were identified by the International Classification of Diseases (ICD) ICD-09 from 1997 to 1999 and ICD-10 thereafter [22]. The following admissions were included: first time AMI (ICD-9: 410; ICD-10: I21.0-I21.3), identified as being primary or secondary diagnoses; stroke (ICD-9: 431,434, 436; ICD-10: I61, I63, I64), identified as being primary diagnoses only; hip fracture (ICD-9: 820 with all subgroups; ICD-10: S72.0-S72.2), identified as being primary or secondary diagnoses. Only the first admission per calendar year per patient was selected. We included hospitals with a minimum of 20 admissions each year during the 5-year period.

Patients were excluded if <18 years for AMI and stroke and <65 years for hip fracture, if the admission was coded as dead on arrival, a non-acute case, readmission or admission for rehabilitation (when identified) and non-first time AMI for AMI patients. Since ICD-9 code 410 covers both first and secondary heart attack, a search for a previous admission to any Norwegian hospital for 410 was made back to 1994 to ensure first time AMI.

### Study sample

Five hospitals were university hospitals, 16 were large, and 45 hospitals were small. A total of 179 293 PAS records of single admissions were identified. We excluded 4 766 (2.7%) records due to missing data, and retained174 527 records from 144 190 patients. For patients with two or more records we established a chain of hospital admissions if time from discharge to readmission or admission to another hospital was ≤24 hours (transferred patients). The use of the inclusion and exclusion criteria resulted in a total of 48 030 AMI patients from 55 hospitals, 47 854 stroke patients from 59 hospitals and 40 142 hip fracture patients from 58 hospitals.

### Mortality measures

Three mortality measures were calculated by counting the number of all-cause deaths as follows:

•Death within 30 days after first day of admission, occurring in-and-out-of hospital, including transferred patients by weighting the outcome to each hospital by the fraction of time (within the 30 day period) spent in each hospital (W30D).

•Death within 30 days after first day of admission, occurring in-and-out-of hospital for patients admitted to one single hospital only (S30D).

•Death within 30 days after first day of admission, occurring in-hospital only (IH30D). For transferred patients, time to death was counted from first day of each admission, i.e. previous hospitals in the chain of admissions counted the patient as survivor.

The various ways of counting are summarized in Table 1. Consider the case of a patient who was transferred from hospital 1 on day 10 and discharged from hospital 2 at day 25, i.e. day 15 in hospital 2. For W30D the outcome of alive is assigned a weight of 10/(10+15) for hospital 1 and 15/25 for hospital 2. For S30D, this patient is not included. For IH30D both hospitals are attributed the outcome of alive. What if the patient stayed 21 days in hospital no. 2 and then died? For W30D, the outcome of alive is assigned to each of the hospitals as the patient died 31 days after start of first admission; hospital 1 is weighted by 10/30 and hospital 2 is weighted by 20/30. This patient is still not included for S30D. For IH30D, the outcome of alive is assigned to hospital 1 whereas hospital 2 is assigned the outcome of death as the patient died 21 days after admission to this hospital.

**Table 1:** How the three different 30-day mortality measures (W30D, S30D and IH30D) account for deaths when place and time of death varies

		**W30D**^**†**^	**S30D**^**‡**^	**IH30D**^**§**^
Place of death	In-hospital, during initial admission	Yes	Yes	Yes
	In-hospital during a subsequent admission	Yes	No	Yes
	Outside hospital	Yes	Yes	No
Start for counting number of days	From Day 0 at the initial hospital	Yes	Yes	No
	From Day 0 at each hospital in the chain of admission	No	No	Yes
Weight attributed to each hospital	(Days at hospital)/ total hospital days)	Yes	No	No
	Unweighted	No	Yes	Yes
Transferred patients included	Yes	Yes	No	Yes

‘Yes’ indicate that the actual place or time of death are included.

^†^W30D: In-and-out-of-hospital mortality within 30 days, attributing the outcome to all hospitals by fraction of time spent in each hospital for transferred patients.

^‡^S30D: In-and-out-of-hospital mortality within 30 days, patients treated at one hospital only.

^§^IH30D: In-hospital mortality within 30 days, for each hospital admission.

### Statistical analysis

Mean, counts and percentages were used to summarize the data. Numbers of deaths were counted for the time intervals ≤30, 31–90 and 91–365 days after start of first admission. The mean length of stay was calculated for each medical condition and for each hospital. Age was categorized as <50, 50–75 and >75 years for AMI and stroke patients and 65–75 and >75 years for hip fracture patients. Seriousness of medical condition was categorized according to the Clinical Criteria Disease Staging (CCDS) system [23] and pooled; for AMI: stages 3.1, 3.2, 3.3 stages 3.4-3.6 and stages 3.7-3.9; for hip fracture: stages 1.1–1.2 and stages 2.3-3.3 [21]. For stroke, seriousness was categorized as either infarction or haemorrhage. Place of death was identified as either during the first admission, death in a subsequent hospital or out-of-hospital death. We recorded when the underlying or any contributing cause of death matched the referral ICD-9 and/or ICD-10 codes.

Unadjusted (crude) mortalities were calculated as the proportion of deaths among all admissions or admission chains according to the definitions of W30D, S30D and IH30D. The adjusted mortalities were estimated by logistic regression models which, in addition to hospital, included the case-mix variables age, sex, and stage of disease. Age was continuous and modelled by B-splines [24]. The hospital regression coefficients were estimated as deviations from the mean of all hospitals [25]. A hospital with higher mortality than the average has a positive coefficient and a hospital with lower mortality than the average has a negative coefficient.

The hospitals were ranked according to mortality by each of the unadjusted mortality measures and by the coefficients from the logistic models. We compared the ranks of S30D and IH30D to that of W30D by the Spearman rank correlation coefficient and by the numbers of hospitals shifting rank. Shifts were categorized as none, minor (1–5 shifts), moderate (6–10 shifts), and major (>10 shifts). Correlations between W30D, S30D, IH30D and length of stay were also estimated. The absolute difference in rank between S30D and W30D and between IH30D and W30D were explored by analysis of variance (ANOVA) for the three hospital categories (university, large, small).

The hospitals were categorized as having high, medium or low mortality: Z-values lower than the 5-percentile (of the normal distribution) identified outlier hospitals with low mortality, Z-values above the 95-percentile identified outlier hospitals with high mortality, medium mortality: remaining hospitals. The association between change/no change in outlier status between S30D and W30D and between IH30D and W30D were explored by Fisher’s Exact tests for the three hospital categories.

C-statistic (area under the ROC Curve) was calculated as a measure of the models’ ability to predict mortality. In general, C-statistic values above 0.7 are considered acceptable [25].

The analyses were conducted using SAS Software, version 9.2 (SAS Institute, Inc, Cary, NC) and R, version 2.11.0 (free software available at http://www.r-project.org/).

## Results

### Patient characteristics

Disease and patient characteristics are summarized in Table 2. The majority of patients was admitted to one hospital only, while 4.8%-6.6% were transferred between hospitals. AMI constituted the largest patient group. These patients had shortest overall mean length of stay (8.6 days), the smallest proportion of females (38.0%) and the youngest patients. The stroke patients had the longest mean length of stay (14 days), half of the patients were females and 56.0% were >75 years. The hip fracture patients had the largest proportion of females (74.2%) and were older (79.9% >75 years).

**Table 2:** Number of hospitals, patient characteristics, time, place and number of deaths for each of the medical conditions

	**AMI**^**∥**^	**Stroke**	**Hip fracture**
Total number of hospitals, N	55	59	58
Small (hospitals/admissions)	34/ 17 994	38/ 19 933	39/ 16 750
Large (hospitals/admissions)	16/ 22 172	16/ 21 223	15/ 18 588
University (hospitals/admissions)	5/ 9 963	5/ 8 925	4/ 7 370
Total number of patients	48 048	47 854	40 142
Transferred patients, n (%)^*^	2 463 (5.1%)	2 293 (4.8%)	2 649 (6.6%)
Mean length of stay (range of individual hospitals), days	8.6 (3.9 - 10.7)	14.0 (6.3 - 25.0)	11.8 (5.6 - 30.4)
Gender, females, n (%)*	18 238 (38.0%)	23 814 (49.8%)	29 801 (74.2%)
Age, n (%)*			
< 50 years	3 888 (8.1%)	1 860 (3.9%)	¤
50 – 75 years	23 993 (49.9%)	19 209 (40.1%)	8 074 (20.1%)¤
>75 years	20 167 (42.0%)	26 785 (56.0%)	32 068 (79.9%)
Time to death within 1 year, n (%)*			
<30 days	9 158 (19.1%)	8 429 (17.6%)	3 140 (7.8%)
31 – 90 days	1 540 (3.2%)	2 175 (4.5%)	2 621 (6.5%)
91 – 365 days	2 837 (5.9%)	3 758 (7.9%)	4 557 (11.4%)
Alive > 1 year, n (%)*	34 513 (71.8%)	33 492 (70.0%)	29 824 (74.3%)
Number of deaths within 30 days (% of deaths within 30 days)			
during first admission	7 980 (87.1%)	6 851 (81.3%)	1 486 (47.3%)
in a different hospital	156 (1.3%)	191 (2.3%)	52 (1.7%)
out-of-hospital	1 022 (11.1%)	1 387 (16.5%)	1 602 (51.0%)
Number of deaths within 1 year, (% of total deaths within 1 year)			
during first admission	8 188 (60.5%)	7124 (49.6%)	1 645 (15.9%)
in a different hospital	167 (1.2%)	212 (1.5%)	69 (0.7%)
out-of-hospital	5 180 (38.3%)	7026 (48.9%)	8 604 (83.4%)
Number of deaths within 1 year caused by referral diagnosis (% of total deaths within 1 year)	7 859 (58.1%)	10 535 (73.5%)	3 863 (37.9%)
Time to death within 1 year for deaths caused by referral diagnosis (% of total deaths within the interval)			
0 – 30 days	6 757 (73.8%)	7 550 (89.6%)	2 383 (75.9%)
31 – 90 days	488 (31.7%)	1 470 (67.6%)	1000 (38.2%)
91 – 365 days	618 (21.8%)	1 537 (41.0%)	482 (10.6%)

^∥^AMI: Acute myocardial infarction.

* % of the total number of patients.

^¤^ Only patients > 65 years old included for hip fracture.

### Time and place of death

After one year, 70.0-74.3% of the patients were alive (Table 2). The proportions of deaths within 30 days were 19.1% for AMI, 17.6% for stroke and 7.8% for hip fracture patients*.* Among the patients who died within 30 days, out-of-hospital deaths occurred for 11.1% (AMI), 16.5% (stroke) and 51.0% (hip fracture). Among those who died within one year, the highest proportion of in-hospitals deaths was for the AMI patients (60.5%) and lowest for the hip fracture patients (15.9%).

### Cause of death

The proportion of deaths with similar referral and cause of death diagnoses was high within 30 days after admission for all three medical conditions (73.8-89.6%, Table 2). Within one year, this proportion was still high for AMI (58.1%) and stroke (73.5%), but considerably lower for the hip fracture patients (37.9%).

### Transferred patients

It was not possible to deduce the reason for transferring patients between hospitals from the data. Few patients were transferred between three or more hospitals (AMI 59 patients, stroke 89 patients, hip fracture 49 patients). For transfers between two AMI hospitals, 29.6% and 25.5% of the transfers were from a small or a large hospital, respectively, to a university hospital (Table 3). The most frequent transfer was from a large to a small hospital for stroke (39.3%) and hip fracture patients (58.2%) (Table 3).

**Table 3:** Number of patients transferred from initial hospital to subsequent hospital, length of stay (days) at initial hospital (LOS1) and length of stay (days) at subsequent hospital (LOS2)

		**Transferred to, hospital category**					
		**Small**		**Large**		**University**	
	From hospital category	n (%*)	LOS1 / LOS2 days, mean	n (%*)	LOS1 / LOS2 days, mean	n (%*)	LOS1 / LOS2 days, mean
AMI	Small	113 (4.7)	6.8 / 6.5	166 (6.9)	4.4 / 6.9	712 (29.6)	4.9 / 5.1
(n=2404^‡^)	Large	383 (15.9)	7.6 / 8.0	113 (4.7)	4.2 / 8.4	614 (25.5)	5.1 / 3.2
	University	129 (5.4)	4.1 / 6.7	165 (6.9)	3.9 / 5.9	9 (0.4)	8.7 / 14.5
Stroke	Small	196 (8.9)	4.9 / 17.6	175 (7.9)	6.3 / 19.0	274 (12.4)	2.3 / 10.9
(n=2204^‡^)	Large	866 (39.3)	6.1 / 13.9	136 (6.2)	7.3 / 22.1	272 (12.3)	1.8 / 9.1
	University	136 (6.2)	7.1 / 20.2	125 (5.7)	7.0 / 28.8	24 (1.1)	30.6 / 35.5
Hip fracture	Small	274 (10.5)	4.7 / 10.1	317 (12.2)	2.8 / 11.7	151 (5.8)	10.7 / 19.5
(n=2600^‡^)	Large	1512 (58.2)	5.5 / 15.1	106 (4.1)	3.9 / 11.0	43 (1.7)	4.3 / 16.4
	University	88 (3.4)	4.3 / 8.9	47 (1.8)	3.3 / 12.1	62 (2.4)	16.2 / 28.9

^‡^Number of patients transferred once.

* % of the number of patients transferred once per disease.

Summarized per medical diagnosis and hospital category.

The mean length of stay at the initial hospital (LOS1) was shorter than at the subsequent hospital (LOS2) for the three medical conditions irrespective of hospital category with the exception of AMI patients transferred from large to university hospitals (mean LOS1=5.1 days versus LOS2=3.2 days) (Table 3). The mean length of stay at the subsequent hospital is considerably longer for all transferred stroke and hip fracture patients as compared to the AMI patients (Table 3).

### Mortality measures

The unadjusted overall mortalities and range for individual hospitals are given in Table 4. The variation between hospitals was large within each mortality measure.

**Table 4:** Overall mortality (%) according to unadjusted measurement W30D, S30D and IH30D, ranges for individual hospitals

	**AMI**^**∥**^	**Stroke**	**Hip fracture**
W30D^†^	18.6 (14.6 – 26.4)	17.4 (13.6 – 26.7)	7.6 (1.6 – 12.8)
S30D^‡^	19.7 (14.6 – 26.6)	17.9 (10.9 – 27.7)	8.1 (0 – 33.3)
IH30D^§^	16.2 (7.0 – 21.7)	14.1 (6.9 – 21.6)	3.6 (1.3 – 6.4)

^∥^AMI: Acute myocardial infarction.

^†^W30D: In-and-out-of-hospital mortality within 30 days, attributing the outcome to all hospitals by fraction of time spent in each hospital for transferred patients.

^‡^S30D: In-and-out-of-hospital mortality within 30 days, patients treated at one hospital only.

^§^IH30D: In-hospital mortality within 30 days, for each hospital admission.

The adjusted mortality measures were highly correlated for AMI (0.82 ≤ *r* ≤ 0.94, Table 5) and stroke (0.78 ≤ *r* ≤ 0.91, Table 4). The correlations between the mortality measures and length of stay were strongest for hip fracture; W30D (*r* =−0.54) and S30D (*r* =−0.35).

**Table 5:** Spearman’s correlations between the adjusted 30-day mortality measures W30D, S30D and IH30D and mean length of stay (LOS)

		**Rank W30D**^**†**^	**Rank S30D**^**‡**^	**Rank IH30D**^**§**^
AMI^∥^ (N=55)	Rank W30D	1.00	0.94	0.90
	Rank S30D		1.00	0.82
	Rank IH30D			1.00
	Mean LOS	0.05	−0.11	0.15
Stroke (N=59)	Rank W30D	1.00	0.87	0.91
	Rank S30D		1.00	0.78
	Rank IH30D			1.00
	Mean LOS	−0.22	−0.22	0.05
Hip fracture (N=58)	Rank W30D	1.00	0.81	0.66
	Rank S30D		1.00	0.49
	Rank IH30D			1.00
	Mean LOS	−0.54	−0.35	−0.02

^∥^AMI: Acute myocardial infarction.

^†^W30D: In-and-out-of-hospital mortality within 30 days, attributing the outcome to all hospitals by fraction of time spent in each hospital for transferred patients.

^‡^S30D: In-and-out-of-hospital mortality within 30 days, patients treated at one hospital only.

^§^IH30D: In-hospital mortality within 30 days, for each hospital admission.

**Figure 1:**
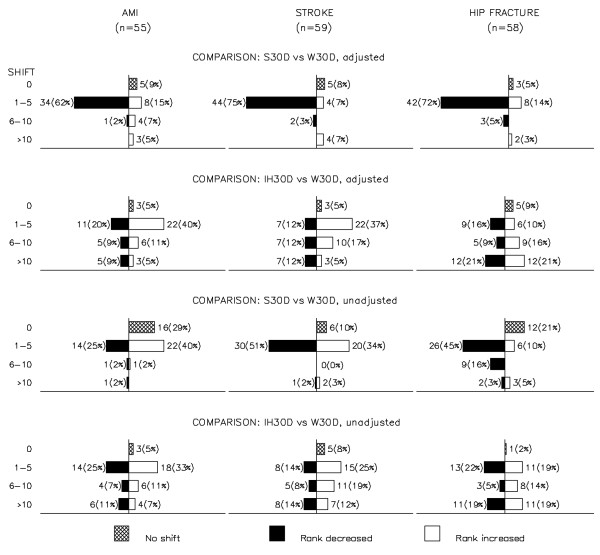
**Number of hospitals shifting rank and direction of shift when comparing the ranks obtained by mortality measures S30D and IH30D versus W30D per medical condition.** Shifts are categorized as none, minor (1–5 shifts), moderate (6–10 shifts), and major (>10 shifts). The top bar on every plot shows the number of hospitals with no shift in rank. The empty bars to the right of the vertical axis show the number of hospitals shifting to better rank (lower mortality) when compared to W30D. The filled bars to the left of the vertical axis show the number of hospitals shifting to lower rank (higher mortality) when compared to W30D.

In Figure 1, back-to-back barplots display the shifts and direction of shift, per shift category, for the hospital ranks when comparing S30D and IH30D to W30D, unadjusted (lower two rows) and case-mix adjusted (upper two rows), per medical condition. The ranking was highly influenced by the method of counting the number of deaths. For the comparisons of adjusted mortalities, no altered rank was seen for 5-9% of the hospitals. Most shifts were minor (77.0%-86%) when comparing S30D versus W30D (upper row 1, Figure 1). For IH30D versus W30D, 14% of the AMI, 17% of the stroke and 42% of the hip fracture hospitals had major (>10) shifts in rank (row 2 from top, Figure 1). Minor shifts in rank were seen for adjusted versus unadjusted measurements.

For AMI, the ANOVA indicated an association between hospital category and the mean absolute rank shift between S30D and W30D (p=0.09). No tendencies were observed for the other medical conditions nor for IH30D versus W30D (0.26≤ p≤0.94).

More hospitals changed outlier status between IH30D and W30D for AMI (18.2%) and stroke (25.4%) than between S30D and W30D (AMI 14.0%; stroke 17.0%), (Table 6). The largest change occurred for one stroke hospital which had low mortality according to W30D and high mortality according to S30D. The remaining shifts were from high or low to medium or vice versa. For hip fracture, no high nor low mortality hospital was identified by S30D whereas nine out of 14 hospitals shifted from high mortality (by W30D) to medium mortality by IH30D. Although non-significant, there was a tendency for an association between change in outlier status for S30D and hospital categories for AMI hospitals (Fisher’s exact test: p=0.06; 0.22≤ p≤0.80 for all other comparisons and medical conditions).

**Table 6:** Number of hospitals per outlier category for the 30-day adjusted mortality measures S30D and IH30D versus W30D

	**S30D**^**‡**^			**IH30D**^**§**^		
	**High mortality**	**Medium mortality**	**Low mortality**	**High mortality**	**Medium mortality**	**Low mortality**
AMI^∥^ (N=55), W30D^†^						
High mortality	8	2	.	8	2	.
Medium mortality	2	38	4	3	37	4
Low mortality	.	.	1	.	1	.
Stroke (N=59) , W30D^†^						
High mortality	12	3	.	11	4	.
Medium mortality	1	26	3	3	23	4
Low mortality	1	1	12	.	4	10
Hip fracture (N=58), W30D^†^						
High mortality	.	14	.	5	9	.
Medium mortality	.	37	.	3	34	.
Low mortality	.	7	.	.	4	3

^∥^AMI: Acute myocardial infarction.

^†^W30D: In-and-out-of-hospital mortality within 30 days, attributing the outcome to all hospitals by fraction of time spent in each hospital for transferred patients.

^‡^S30D: In-and-out-of-hospital mortality within 30 days, patients treated at one hospital only.

^§^IH30D: In-hospital mortality within 30 days, for each hospital admission.

The C-statistics were acceptable for the various mortality measure models (ranges 0.726-0.729, 0.700-0.713 and 0.678– 0.694 for AMI, stroke and hip fracture, respectively).

## Discussion

This study used data that included time, place and cause of death for patients admitted for AMI, stroke and hip fracture to all Norwegian hospitals during a 5-year period. We compared case-mix adjusted hospital mortality measures, based on in-and out-of-hospital deaths for patients admitted to one hospital only (S30D) and in-hospital deaths (IH30D) to that of in-and-out-of-hospital deaths accounting for transferred patients (W30D). Major shifts in hospital ranking and outlier detection occurred.

### Time and place of death

Independently of place of death, the proportion of deaths within the standardized follow-up period of 30 days was considerably lower for the hip fracture patients compared to AMI and stroke patients, in accordance with previously reported studies [12,19,26,27]. For diseases with a high proportion of deaths within 30 days, such as AMI and stroke, only minor changes might be expected in the hospital ranking and outlier status when comparing in-hospital deaths (IH30D) to the measures accounting for in-and-out-of hospital deaths (W30D and S30D) [26]. However, as much as 14%-17% of our hospitals had a major shift in rank for IH30D compared to W30D. Also, the change in outlier status was much higher than we expected for this comparison (AMI: 18.2%; stroke: 25.4%). This might be due to a fairly high proportion of out-of-hospital deaths within 30 days for the two patient groups (AMI: 11.1%; stroke: 16.5%). For hip fracture, the changes in shifts were much larger (42%) and the change in outlier status was also high (27.6%). This might be expected considering the lower short term mortality for these patients and the very large proportion of out-of-hospitals deaths (51.0%).

Follow-up care is important for patient outcome [11,28,29]. Variation in quality of follow-up care may explain some of the difference between in-hospital mortality and in-and-out-of-hospital mortality within 30 days. For hip fracture, the negative correlation between length of stay and W30D and S30D indicates a tendency towards better survival with longer hospital stay. This tendency was weaker for stroke and not present for AMI.

### Cause of death

For deaths within 30 days, the referral diagnosis was given as the underlying or contributing cause for more than 73% of the patients. For deaths during 91–365 days, the proportions were lower – especially for hip fracture. It is well-known that identifying the cause of death may be difficult. Accordingly, including deaths caused by the patient condition or treatment procedures only, may conceal the effect of low quality care resulting in patient death arising from other immediate causes [18]. We therefore recommend inclusion of all-cause deaths.

### Transferred patients

For many patients the episode of care includes more than one hospital. Transferral practices can reflect characteristics of the hospitals, as for instance small hospitals sending seriously ill patients to more specialized hospitals for advanced treatment. In addition, some conditions necessitate a rehabilitation period that involves sending patients to another hospital. Our data show high proportions (>50%) of AMI patients sent from small and large hospitals to university hospitals. The likely reason is that advanced treatments (e.g. percutaneous coronary intervention (PCI) or coronary-artery bypass grafting (CABG)) were performed at the university hospitals and at a few of the large hospitals, thus leading to transfer from small hospitals. For stroke and hip fracture, the most frequent transfer was from a large to a small hospital. This may be due to patients admitted to a large hospital for the initial treatment and subsequently transferred to a small hospital for follow-up and rehabilitation. The mean length of stay at the second hospital is considerably longer for stroke and hip fracture patients as compared to the AMI patients. This may indicate the need for a longer follow-up period for stroke and hip fracture patients. Transferred patients may also present more serious condition necessitating a longer period of medical treatment.

In Norway, much effort has been put into centralization of specialized patient treatment and therefore, the transfer rate has increased over the past few years. Including or excluding in-transferred patients has previously been shown to be important for hospitals treating patients with AMI [15,20,30]. This may be explained by a high transfer rate (15%). Our data had low transfer rates (<6.6%). We would thus expect larger differences between S30D and W30D when applying newer data for exploring the association between mortality and transfers and their impact on hospital performance measurement.

We are not aware of research that provides a strong theoretical and empirical basis for attributing the outcome for a single patient to several contributing health care providers. If one hospital cares for the patient in a more critical and life-threatening stage it might be tempting to assign the outcome to this hospital only. However, in the perspective of quality surveillance all hospital stays are important. Thus, there should be some sharing of outcome. The weighting approach (W30D) avoids double counting and bias due to omitted hospital admissions. However, there may be various ways of weighting. Consider a patient who receives one-day extensive critical care at a university hospital and is subsequently transferred to a small hospital for nine days follow-up care. Our approach weights the outcome by 0.9 for the small hospital and 0.1 for the university hospital. Conceivably, the weights could have been exchanged, or the hospital providing the most critical care should always be weighted more (0.5 or more?) and the remaining weight distributed among the other hospitals. This would require a detailed break-down of the care process into diagnostic procedures and interventions as well as considerations of the organization of care. A quantitative extension of the qualitative research of e.g. Bosk et al. would be welcome [31]. Our approach to bias reduction has the virtues of simplicity and transparency. In the absence of any theoretical or empirical guidance, we regard our weighting scheme as the least unsatisfactory of the readily available alternatives.

Small hospitals are thought to have larger variation and thus change status compared to larger hospitals when counting the number of deaths in various way [15,32]. The influence of hospital size on the difference between mortality measures was minor in our data. We found an indication of a difference between the hospital categories when comparing S30D and W30D for the AMI hospitals. This may be due to one university hospital with no local hospital function receiving a large proportion of in-transferred patients from a large number of small hospitals. For hip fracture no outlier hospitals were found by S30D and only 5 out of the 14 high mortality hospitals were detected by IH30D. These results suggest that important variation between hospitals are not identified for mortality measures when including patients treated at one hospital only.

### Strengths and limitations

The unique PIN enabled the merging of data from different hospitals and the official registries. Thus, the entire chain of admissions for a patient was accounted for as well as time, place and cause of death. Only 0.85% of the records were excluded because of an invalid PIN, mainly due to patients who are non-permanent residents and thus are assigned a temporary PIN upon hospital admission. Our data covered all Norwegian hospitals and admitted patients for the three medical conditions.

The importance of coding and consequences for hospital ranks and outlier detection has been reported [13]. Variation in diagnostic coding practice may explain differences in mortality between hospitals. Another concern has been that the patient case-mix may be incorrectly represented. Insufficient or absent adjustment for case-mix or even different ways for treating the case-mix in the calculation of mortality, may cause bias in the actual hospital ranking and outlier detection [11,13,32]. We have included three case-mix variables that are important for prediction of mortality [11,32]. The similar profiles for shift in rank for adjusted and unadjusted calculation of W30D, S30D and IH30D indicate little impact of case-mix for the comparison of measures. Extending our calculations to include more case-mix variables, e.g. more medical and socio-economic information, is subject of further research.

Presenting hospital performance by use of ranking lists has been criticized [5,8]. We found the shift in rank useful for the comparisons of the mortality measures. The change in outlier status confirmed the large variation in hospital performance when using different mortality measures. This demonstrates the importance of how we count for mortality measures.

## Conclusions

Mortality measures based on in-hospital deaths alone or measures excluding admissions for transferred patients, can be misleading as indicators of hospital performance. We recommend the use of case-mix adjusted morality based on in-and-out-of-hospital deaths within 30 days. We propose to attributes the outcome to all hospitals by fraction of time spent in each hospital for patients transferred between hospitals to reduce bias due to double counting or exclusion of hospital stays.

## Competing interests

The authors declare that they have no competing interests.

## Authors’ contributions

JCL was project leader for the data collection and the report which formed the basis for the present work. All authors conceived the design and content of this paper. DTK and JH performed the analysis. DTK drafted the first version. All authors contributed to revised versions, read and approved the final manuscript.

## Pre-publication history

The pre-publication history for this paper can be accessed here:

http://www.biomedcentral.com/1472-6963/12/364/prepub
